# Reaching conceptual stability by re-articulating empirical and theoretical work on affordances

**DOI:** 10.3389/fpsyg.2024.1283168

**Published:** 2024-06-14

**Authors:** Roman Malo, Yannick Prié

**Affiliations:** ^1^Nantes Université, Laboratoire de psychologie des Pays de la Loire, LPPL, UR 4638, Nantes, France; ^2^Nantes Université, École Centrale Nantes, CNRS, LS2N, UMR 6004, Nantes, France

**Keywords:** affordances (ecological psychology), direct perception, empirical methodology, Gibson, 4E cognition

## Abstract

Theoretical developments on affordances have proliferated, resulting in a lack of conceptual stability and a potential compromise in scientific validity. However, affordances should not be discarded, given their centrality in post-cognitive theories and their widespread reuse across various research domains. Empirical research on affordances remains sparse, out of sync with theoretical advancements, and thus unable to contribute effectively to scientific progress due to its disarticulation with theoretical work. That is why re-articulating theoretical and empirical investigations on affordances is needed to pave a more fruitful path for the concept’s advancement. To accomplish this objective, emphasis must be placed on empirical research, leveraging recent theoretical propositions and devising corresponding empirical methodologies. The proposed requirements and framework represent a step in this endeavor.

## 1 Introduction

Gibson (1977) introduced the concept of affordance more than 40 years ago. In doing so, he challenged the dominant paradigm at that time, by asserting that perception was not the passive reception of stimuli but rather an integrated perceptual action that takes place within a dynamic interaction between the subject and the environment. In his theory of direct perception, Gibson proposed that perception is direct -in the sense of unmediated-, that it is an active process -meaning the perceiving individual is not passive-, and that perception and action are tightly connected. Gibson also focused on an “ecological” proposition for perception, with two main claims: there exist “ecological niches” specific to animals, and what is perceived is what the environment directly affords to animals for behavioral purposes (mainly surviving goals). Affordances were introduced as a neologism to denote what was directly perceived in the world. “To see things is to see how to get about among them and what to do or not do with them” (Gibson, 1986, p. 223).

Over time, the concept has revealed itself as a central element in modern theories of subject-environment coupling, that it would be, rather naturally, for ecological psychology, for enactivism, or overall for 4E cognition. In addition, the ability of affordances to describe certain elements of the activity of individuals in their environment, namely what the environment affords to them, made them an appealing concept for other theoretical fields. As a consequence, affordances also became a cornerstone for a diversity of fields beyond perception sciences, as they were integrated among others into psychology (e.g., Tucker and Ellis, 1998), design (e.g., Norman, 1999), or architecture (e.g., Rietveld and Brouwers, 2017). Yet, despite this undeniable theoretical prosperity, we identify two issues that may put them at risk of losing their scientific value.

A first issue is the constitutive ambiguity of affordances. Since its origin, “affordance” has remained an elusive neologism, leaving researchers puzzled about its status (Luyat and Regia-Corte, 2009). This can particularly be seen in the discussions about its dispositional (e.g., Caiani, 2014) or relational nature (e.g., Chemero, 2003; Rietveld and Kiverstein, 2014), or about the roots of the perception of affordances, namely the sensory organs or the organism as a whole (Read and Szokolszky, 2020). Such conceptual ambiguity sparked a persistent debate within scientific literature about how to precisely understand what affordances are. Despite most authors claiming to fall in line with the original Gibsonian proposal, their use of the term and their conceptual proposals do not always correspond to a shared conceptualization, or to the adhesion to an ecological, direct theory of perception. As we will see further down, there is currently no theoretical consensus on what affordances are, and no theoretical solution seems at hand.

We witness not only the absence of theoretical consensus, but also a second issue that we propose to call a *disarticulation* between theoretical and empirical research. This disarticulation is grounded in another originary ambiguity in early affordance theory: the lack of instructions on how to empirically assess them, which led to vastly different empirical approaches. For instance, “classical” approaches seek to “measure” affordances from a primarily body-scaled perspective, using metrics derived from experimental psychology, mostly in laboratory settings. On their side, “contextual” approaches view affordances as socially situated and study them in “context,” favoring observation in everyday life situations, and the use of exploratory methods to account for subject-environment coupling. More generally, conceptual differences between researchers have led to diverging empirical methodologies (Heft, 2003), which results are hardly comparable. Empirical work appears scattered, empirical researchers lack common ground and community, which prevents the accumulation of coherent empirical material at scale. Moreover, despite its successes, classical empirical research hardly led to theoretical evolutions, while contextual empirical research seems to have limited theoretical ambitions. On their side, theorists appear to have had little interest in empirical data, and to not seek for an empirical validation of their proposals. The disarticulation may also be worsening, feeding and reinforcing itself, because theoretical productivity has not been matched by related empirical efforts, and because empirical research appears underrated, which is a compounding factor.

The concept of affordance lacks conceptual stability, and the disarticulation between empirical and theoretical work prevents clarification, or settlement on theoretical disputes (Chong and Proctor, 2020). This leads to difficulties in achieving a consequent critical academic community that would be necessary to sustain a fruitful empirical and theoretical research dynamic, with coherent lines of research, based on common theoretical grounds and associated empirical methods. As a consequence, and despite their undeniable attraction and significance across various fields of research, affordances may lose their status as a scientific concept. This is why a re-articulation between empirical and theoretical work is crucially needed, that would help reaching conceptual stability.

Such re-articulation should go in two directions: theoretical work must systematically seek empirical data to support its propositions, and develop them as empirically verifiable, while empirical work needs to develop innovative empirical methodologies that match theoretical work. For this, we propose that empirical research should first be based on a minimal shared foundational definition of affordances as “action possibilities,” emphasizing its integration into the theory of direct perception. It also seems necessary to acknowledge the importance of recent theoretical contributions to affordance theory, particularly those of a relational lineage (e.g., based on coupling, subject-environment relationship, cultural and social aspects), and we propose some requirements in that direction, namely to take into account inter-individual variability, study lived experience of affordances, and multiply indicators and proxies for accessing them through mixed methods in ecological contexts.

In the following development we first give an overview of the main theoretical works on affordances, and highlight the theoretical impasse in which the concept may be blocked. We then present the two main lines of empirical studies of affordances, and discuss their shortcomings and the overall problem with the disarticulation between theoretical and empirical research. Finally, we propose a step toward re-articulating empirical and theoretical work by presenting several requirements for empirical research, and the associated guidelines for building adapted empirical methodologies.

## 2 Theoretical studies of affordances

### 2.1 Affordance as an evolving concept struggling to stabilize

#### 2.1.1 The fundamental debate on the nature of affordances

Gibson defined affordances as follows: “The affordances of the environment are what it offers the animal, what it provides or furnishes, either for good or ill” (Gibson, 1986, p. 127). Such a proposal marks a turning point in perception research by proposing an ecological perspective (Neisser, 1990; Dotov et al., 2012). Gibson’s main claim is that animals have a direct perception of the world, breaking with the idea of perception as representation building from the inputs of the senses, and limiting the place of top-down processes.^1^ Since then, affordances have been the subject of a great deal of research rooted in an alternative vision of the classic representational models of cognition (e.g., Kono, 2009; Rietveld and Kiverstein, 2014; van Dijk et al., 2015). The concept continues to evolve, generating a dense theoretical debate, but also a great deal of confusion (Chouinard and Davis, 2016; Osiurak et al., 2017).

An immediate observation when reviewing the literature is the polysemy of the term, already highlighted by Oliver (2005). As Chong and Proctor (2020, p. 120) remark, “there is no singular definition of affordances and […] discussions of the concept do not strictly adhere to the theoretical work conducted by Gibson.” Many authors have indeed taken a lot of different positions over the years, and there is no consensus. Such difficulty to build a single shared definition might be illustrated by an important debate on the nature of affordances, between the so-called dispositional and relational approaches.^2^

The “dispositional interpretation of affordance” (Caiani, 2014) considers that affordances are dispositions -or potentials-, constituted by presently occurring anchoring properties of the environment that are not made actual until in presence of the right complementary disposition (e.g., salt can dissolve in water, and its “solubility” is only effective when it meets a liquid) (Turvey et al., 1981; Turvey, 1992; Reed, 1997; Silva et al., 2013). This approach has been mainly developed by Turvey (1992), Scarantino (2003) or later on by Wilson (2018) or Golonka and Wilson (2019). It suggests that there are invariants in what invite individuals of a given species to act and that affordances have intrinsic characteristics. The argument is mainly centered on animal behaviors such as climbing, catching or feeding, which are based on skills that are globally always present for animals. The majority of dispositional orientation authors relate to Gibson’s heritage.

The relational approach considers that affordances are properties of the animal-environment relationship (Chemero, 2003; Stoffregen, 2003, 2004; Rietveld and Kiverstein, 2014). This approach has its origins in the work of various authors who have extended Gibson’s ideas by emphasizing the role of cultural and historical context, bodily experience, individual action possibilities, and the environment shaping the perception and interpretation of affordances (Heft, 2003; Stoffregen, 2003; Costall, 2012). An example is the concept of Affordance 2.0 proposed and developed by Chemero (2001, 2003, 2011). “Consider the interaction over time between an animal’s sensorimotor abilities […] and its niche, that is the set of affordances available to it. […] The key point here is that affordances and abilities are not just defined in terms of one another […] but causally interact in real time and are causally dependent on one another” (Chemero, 2009, pp. 151–152). Here Chemero suggests that an affordance is an element of both the environment and the subject in a dynamic (and therefore potentially changing) relational perspective.

Choosing one approach over the other can raise tricky questions, particularly with regards to the basic elements of the definition: if an affordance is a disposition, then does it exist even when the complementary property is not present? How can we explain the perception of specific dispositions? And if it is a relation, how can one learn a new ability if one cannot perceive the affordance that supports it until one performs the action corresponding to that ability? How can we precisely describe affordance-as-relation characteristics? Authors such as Golonka and Wilson (2019) consider the dispositional approach as the best starting point, emphasizing that theoretical models focused on “relationships” instead of dispositions face an explainability problem: “Unfortunately, these theories lack a mechanism by which these relational affordances might be perceived” (p. 241).

#### 2.1.2 Theoretical evolutions of affordances

The debates around the nature of affordances led to proposals in order to develop and to shed new understanding of the concept.

Rietveld and Kiverstein (2014) proposed an enactive and ecological perspective to reduce the tension by considering affordances simultaneously as relations and dispositions. Affordances are then both related to the environment and the abilities of life forms whose members can “potentially detect”^3^ them (Rietveld and Kiverstein, 2014, pp. 338). Baggs and Chemero (2019) suggested that the two stances (disposition or relation) are not radically opposed, but are in fact two different levels of understanding, or reading, affordances. A dispositional understanding seems more suited to the *Habitat* of a general life form of a specific species (e.g., humans, birds), a relational one to the *Umwelt* of a specific individual having specific abilities.

Such attempts to find compromises are accompanied by multiple conceptual evolutions, the relation vs. disposition questioning always appearing as a background. Researchers who defend affordances as relations have generally broadened the concept to integrate social, linguistic, mental, etc. considerations (Reed, 1997; Chemero, 2011; Rietveld and Kiverstein, 2014; Wilson, 2018). Researchers focusing on the dispositional nature of affordances have been mainly focusing on finding and describing those dispositions.

We can categorize the theoretical evolutions proposals into extensions of the concept of affordance, and additions of new related concepts and theories.

First, the concept of affordance has been extended, with the appearance of “mental affordances” as proposed initially by Scarantino (2003) and then by McClelland (2020), “social affordances,” “intellectual affordances,” “cascading affordances” (about the terminology linked to affordance, see Overhill, 2012) or lately, “cognitive affordance” in Jorba’s (2020) work. For cognitive and mental affordance proponents, the experience of thinking consists, at least partly, in “seeing” what one can do with one’s thoughts and how to continue from what one has already thought. Other extensions are based on the understanding that affordances are embedded in our social and cultural practices (Rietveld et al., 2013; Rietveld and Kiverstein, 2014, pp. 329). In such a view, an affordance belongs to a cultural and social niche, and is no longer simply a physical action possibility based on physical dispositions. Such a proposal was already present in the 1990s’ literature (e.g., Valenti and Gold, 1991), but that of Rietveld and his colleagues is more developed and marks a turning point. Indeed, it is now accepted to consider sociocultural embedment in the definition of affordances, although this is not a direct Gibsonian legacy (Ramstead et al., 2016; Chong and Proctor, 2020; Dings, 2020). Ramstead et al. (2016) continue in this direction by proposing a distinction between “natural” and “conventional” affordances. Natural affordances depend on individual physical abilities while conventional affordances shape individual action possibilities based on norms and social practices.

Second, affordance theory has undergone significant development and refinement in recent years, with several additions, mostly from a relational perspective. Affordance 2.0 is a theoretical framework that emphasizes the interactive relationship between sensory-motor abilities and the environment, suggesting that they influence each other in retro-active loops (Chemero, 2003, 2009). The concepts of “Landscape of Affordances” and “Field of Affordances” have been proposed by Rietveld and Kiverstein (2014) to highlight the diversity of affordances in a world of possibilities, and to recognize the role of the subjects in shaping the landscape through their activities. Shaw et al. (2019) proposed the concept of “token” to understand how affordances can change over time by distinguishing between affordances as abstract types and affordance-tokens as concrete cases that are situated in space–time for individuals. The concept of “solicitation”^4^ was also introduced as part of a research focus on understanding how different individuals are differently invited to act (Dings, 2020). Solicitations allow us to recognize that all surrounding possibilities do not necessarily become invitations (de Vignemont, 2015, p. 8), and to take into account the phenomenological dimension of “responding” to these invitations: “being invited generates a tension of something that stands out to be done” (Bruineberg et al., 2019, p. 5234).

The variety of these developments illustrates the fact that many questions about the nature of affordances remain, which can be seen as a positive sign regarding the vitality of the field, but also as a negative sign linked to the lack of stability of the concept and the difficulty to reach a theoretical consensus. As a consequence, to date, the meaning of the term is neither stabilized nor shared. For Chong and Proctor (2020) it remains “underdeveloped,” while Baggs and Chemero (2019, p. 9) provocatively observe that “no one knows what affordances are anymore.”

### 2.2 Where is theoretical affordance research going?

Three elements further obscure the debate. Firstly, the lack of consensus may not come as a surprise, because the concept has suffered from ambiguity since its proposal by Gibson, up to the present day (Luyat and Regia-Corte, 2009). Heras-Escribano and De Pinedo-García (2018) highlight the fact that Gibson’s proposition was highly innovative, as it challenged two classical positions in research on subject-environment relationship: the objective/subjective dichotomy of perception and the representationalist view of world’s meaning.^5^ As revolutionary as it was, the proposal was also ambiguous and did not clearly define the nature of affordances, what makes them up, and overall did not clearly identify the limits of direct perception. It was legitimate to think that time would clarify this ambiguity, but this is not the case. Some researchers even argue that the only way to overcome such inherent ambiguity is to adopt a dispositional perspective (Golonka and Wilson, 2019).

Secondly, current theoretical research on affordances is clearly influenced by the relational perspective, which gains importance. Researchers following this perspective have been enriching the notion by attributing additional characteristics to it or adding ancillary notions, making it (even) more difficult to grasp. Moreover, this enrichment took place on the fringes of a debate on how to read Gibson, and on what can or cannot be produced as a legacy. Indeed, each side has arguments as to whether or not Gibson was in a dispositional or relational state of mind (Luyat and Regia-Corte, 2009). Disposition-oriented researchers claim that they directly follow Gibsons’ dispositional ideas, they promote the Gibsonian theory of direct perception that includes affordances, and the close link between these and the biomechanics qualities of the individuals. Relation-oriented authors use the subjective/objective indistinction argument proposed by Gibson: “An affordance is neither an objective property nor a subjective property; or it is both if you like. An affordance cuts across the dichotomy of subjective-objective and helps us to understand its inadequacy” (1986, p. 129) to justify their affiliation, which allows them to further extend the concept in this direction. Whatever Gibson actually meant, it seems nevertheless that the proposals from the relational view of affordances, which is gradually prevailing, are moving away from Gibson. For instance, mental affordances would lead to “mental” and not physical actions, and considering social affordances requires symbolic information (Bruineberg et al., 2019). The fact that fundamental research on affordances may be departing from the Gibsonian legacy adds to the instability, because this is not clearly stated: most authors remain elusive on the matter, mentioning Gibsons’ original definition in the introduction, not discussing the nuances of the approach, nor their differences with his theoretical work (Chong and Proctor, 2020, p. 119).

Lastly, many authors have been borrowing the concept of affordance to use it in a growing body of research fields not directly related to perception research. A non-exhaustive list would at least contain: phenomenology (Dreyfus and Dreyfus, 1999), neuroscience (Cisek and Kalaska, 2010), sociology (Schatzki et al., 2000), sports science (Araújo and Davids, 2016); linguistics (Gumperz and Levinson, 1996), cognitive psychology (Tucker and Ellis, 1998), architecture (Maier et al., 2009; Rietveld and Brouwers, 2017); engineering (Effken and Shaw, 1992; Duchon et al., 1998; Chemero and Turvey, 2007; Rome et al., 2008); engineering design and industrial design research (Brown and Blessing, 2008; Galvao and Sato, 2008). Affordances can also be found in music (Krueger, 2011); anthropology (Ingold, 2018); design (Norman, 1999, 2015); and artificial intelligence (Horton et al., 2012). All these borrowings clearly show the attractiveness of the concept and its potential theoretical usefulness in various domains. Yet the lack of theoretical rigor in the use and the associated elaborations do not help to develop conceptual clarity (Chong and Proctor, 2020), and add to the confusion about what affordances really are. Also, they imply that current research on affordances is less and less concerned with Gibsonian legacy.^6^

If we focus back on “core” research on affordances, namely perception theory and cognitive science, we can only note that researchers have explored several theoretical avenues to resolve the ambiguity of the concept and develop its usefulness, in a constant clarification attempt. However, the resulting conceptual instability can be associated with the major threat of losing scientificity. Indeed, as suggested by authors such as Popper (2002) or Kuhn (1962), for a concept to be scientific, it must have a specific identity, and a definition of what it is and what it is not by exclusion. With that regard, the difficulty to maintain a stable and unequivocally interpreted definition of affordances over time, the various and inconsistent definitions, and overall the lack of identity may be a sign of the difficulty, if not the impossibility, of attaining scientificity, and fruitfulness in the sense of Kuhn, i.e., generate further coherent knowledge, experimentation, and overall progress. This further hinders the possibility of building a strong community and contributing to the development of shared knowledge, another obstacle to scientific viability.

### 2.3 The need for a more productive course for affordance research

Despite their undeniable appeal, affordances may not be as fruitful as expected for a productive exploration of subject-environment relationships. Some, like Oliver (2005), consider that the concept has been problematic since its inception: “Even in Gibson’s sense, the term is problematic” (p. 412), going as far as suggesting it should be abandoned. We believe, however, that affordances should not be canceled, because (1) they have the potential to act as an important boundary object between different fields, and (2) they have an important role to play in contemporary cognitive science theories such as 4E cognition, and overall psychology (Heft, 2003). Let us elaborate.

On the one hand, a lot of research fields use affordances to account for possibilities of action in the subject-environment dynamic. This is rare enough to be worth mentioning, and it seems to us that the concept is a natural candidate for becoming an abstract boundary object between disciplinary fields. Indeed, boundary objects encourage exchanges and collaborations between different communities, they are “both plastic enough to adapt to local needs and constraints of the several parties employing them, yet robust enough to maintain a common identity across sites” (Star and Griesemer, 1989). However, in order to qualify as such, to become a concept common to several fields -and not just a mere, possibly metaphorical, borrowing from the sciences of perception- affordances must meet a minimum stability criterion, and have a shared basic definition. The search for “stable” constituent characteristics of affordances therefore represents a difficult but necessary challenge if they are to become the object of dialog between the concerned research fields.

On the other hand, affordances may be a central concept for 4E cognition, an approach which aims to unify post-cognitivist theories by rethinking cognition, considered as Embodied, Embedded, Extended, and Enacted (Carney, 2020). Researchers following that approach outline the importance of assessing the role of the *body* in cognition, as well as that of *external* objects. Cognition can also *extend* the limits of the individual, and there is a coupling between the individual and the environment, perception being an active rather than a passive process, action *enacting* a world as an environment one can act in. Many of these ideas resonate with relational affordance theories. For Newen et al. (2018, p. 9), 4E cognition is deemed relational in nature, and might even be “affordance-based.” Hampson et al. (2021) argue that the 4E cognition approach necessarily relies on the notion of affordance, as it adopts the Gibsonian postulate that “perception is a function of organism-environment fit, enacted via the sensory detection of immediate action possibilities (aka affordances)” (pp. 514–515). Rietveld et al. (2018, p. 42) point out that the concept of affordance is indispensable if we wish to understand the embodied mind, as they provide a simple way to talk about how behavior and context are codependent, and how the coupling between humans and their environment occurs: any possibility of action (or affordance) enables people to act and create the environment to act in. This can be thought of at two levels, that of the instantaneous dynamic coupling, and that of the development of the coupling, i.e., of abilities. The individual creates a world of possibilities for action, and at the same time, these affordances influence the individual state of action readiness, abilities and cognitive organization (Rietveld et al. 2018, p. 43).

Whether it is to play the role of a boundary object between various disciplinary fields, or to become a building block of post-cognitivist theories, the concept of affordance is much needed. But it also needs to stabilize, i.e., to reach a minimal definition that remains sufficiently stable for a sufficiently long period of time for researchers to have productive exchanges within a community. It seems to us that attaining this objective can not be based solely on theoretical remodeling, but must involve empirical work. In the following section, we focus on the current state of empirical work on affordances, exploring two main approaches.

## 3 Empirical studies of affordances

Heft (2003) highlighted three specific “trends” in research on affordances “that participate in bracketing or delimiting affordances” (pp. 173–174). He proposed that researchers’ interests may vary between (1) the physical-body attributes of an individual; (2) perceptual learning as the driver of changes in how possibilities are seen (e.g., Gibson and Pick, 2000); or (3) the intentionality of the perceiving act. By doing so he suggested that the way researchers study affordances empirically differ depending on the way they define them.

With regards to empirical research on affordances, and applying a similar way of thinking, it seems to us that there are two main approaches (Table 1). “Classical” approaches^7^ inherited from experimental psychology are mainly based on a dispositional thinking, using metrics to measure affordances with classic scientific proxies. More recent “ecological” or “contextual” studies^8^ are based on relational thinking, aiming to account for affordances in real life situations with exploratory methods based on a diversity of data. We will now explore these two trends, before examining if current empirical works on affordance research could help stabilizing the concept.

**Table 1:** Overview of empirical research on affordances.

Type of empirical studies	Exemplary topics and associated works	Theoretical framework	Experimental setting	Method of data collection and analysis
Classical experimental studies of affordances	**Ascending slopes / traversability of surfaces (children):** Gibson and Walker (1984), Gibson et al. (1987), and Adolph et al. (1993) **Perceiving reachability**: Carello et al. (1989) **Surfaces walkability:** Kinsella-Shaw et al. (1992) **Gap crossability**: Burton (1992, 1994), and Burton and McGowan (1997) **Passing under a barrier:** van Der Meer (1997) **Contextual modulation of responsiveness to affordances (kitchen):** Wokke et al. (2016) **Crossing ability (firefighters):** Petrucci et al. (2016) **Dutch-crossing ability (in VR):** Gagnon et al. (2021)	**Definition:** “The affordances of the environment are what it offers the animal, what it provides or furnishes, either for good or ill” (Gibson, 1986, p. 127) **Affordance nature:** Mainly dispositional Body-scaled, sensori-motor **Added notions:** One affordance at the time	Mainly in laboratory	**Method(s):** Mainly third person perspective **Focuses:** proxies of affordances (reaction time, ratio between size/possibilities, optimal and minimum threshold); physiological measures such as EEG
Contextual exploratory studies of affordances	**Psychiatry and psychopathology influence on affordances:** de Haan et al. (2013) **Sports and improving human-environment interaction in performance:** Seifert et al. (2014) and Rochat et al. (2019) **Architecture and user behavior**: Withagen and Caljouw (2016) **School setting and children in free-play situation:** Bjørgen (2016), Lerstrup and van den Bosch (2017), and Sando and Sandseter (2020)	**Definition:** Affordances are possibilities to act in an environment, embodied in socio-cultural practices (Rietveld and Kiverstein, 2014) **Affordance nature:** Mainly relational **Added notions:** Landscape of affordances	Mainly in daily-life	**Method(s):** First and third person perspective First person: Semi-structured, self-confrontation, explicitation interviews, think-aloud Third person: questionnaires, observations, videos **Focuses:** individual experience/subjective proxies of affordances

### 3.1 Classical empirical laboratory work on affordances

#### 3.1.1 Measuring how people perceive affordances

The majority of classical experimental studies of affordances aim primarily at reaching and “measuring” them empirically. In this section we present some representative works, without aiming to be exhaustive. The associated experimental frameworks are largely influenced by the original dispositional and body scaled sensorimotor approach of affordances. Experimental designs propose participants with objects in the world that are not necessarily perceived *per se* (e.g., stairs, doors, chairs), but through their associated physical possibilities of action / affordances (e.g., going up, passing, sitting).

Warren’s (1984) experiment, better known as the “staircase” experiment, is a reference for anyone interested in measuring how people perceive affordances (Şahin et al., 2007; Luyat and Regia-Corte, 2009). Warren proposed to look for critical and optimal points at which a step is considered “mountable.” He showed that one’s judgment of one’s ability to climb a stair step is not determined by its overall dimension, but by the ratio between the step’s height and one’s leg length, based on one’s own bio-mechanical dimensions. In the same path, other experiments have proposed to study affordances related to the “catchability” of an object (Carello et al., 1989), the “crossability” of a ditch (Burton, 1992, 1994), or the “passability” under a barrier (van der Meer, 1997).

Eleanor Gibson studied the way in which children perceive affordances by observing their exploration behavior when faced with surfaces of different rigidity or deformability (Gibson et al., 1978). In another research, she showed that accessibility changes along with children’s development and their tendency to overestimate their ability to climb slopes (Gibson and Walker, 1984; Adolph et al., 1993; Gibson, 2000). Later, Kinsella-Shaw et al. (1992) studied the slopes that adults consider to be “walkable.” In this experiment and its replications, they showed that the perception of the maximum slope on which it is possible to walk (affording “walkability”) corresponds closely to the actual maximum slope that allows normal walking (i.e., before the walking movement is distorted).

In 2016, Petrucci et al. examined how firefighters perceive “obstacle passability” while wearing gear that alters body dimensions. They showed perceptual errors, mainly the overestimation of crossing ability with the equipment, and that more experienced firefighters had reduced errors. Wokke et al. (2016) explored affordances in daily life (cooking in a kitchen), investigating how specific variables influence affordance perception, and object/context congruence. Their study revealed that the environment affects affordability and responsiveness, measured through electroencephalographic recordings, with cooking utensils perceived as more attractive in a kitchen than in a workshop.

These classical experiments continue developing with Virtual Reality (VR) (Pointon et al., 2018; Wu et al., 2019; Gagnon et al., 2020). Indeed, VR technology allows the development of more complex and controllable environments, enabling the study of affordances in specific situations that are difficult to reach in reality. These studies extend classical experiments such as the ditch-crossing ability (Gagnon et al., 2021) or the possibility of using a staircase (Asjad et al., 2018). It appears that the results are similar to those obtained in reality (Geuss et al., 2010; Lin et al., 2015; Stefanucci et al., 2015; Bhargava et al., 2020), the exception being when the degree of “immersiveness” of the VR environment is insufficient to match reality (Ebrahimi et al., 2018; Gagnon et al., 2021).

#### 3.1.2 Characteristics of classical experimental empirical research

An overview of this “classical” line of empirical research on affordances reveals some common features in the understanding of affordances and the methodologies used.

1. Research is mainly oriented toward sensory-motor “affordances.” Most studies are based on the original definition of affordances. They focus on physical actions, which are considered directly feasible depending on the anthropometric or bio-mechanical (body-scaled) characteristics of the participants (Şahin et al., 2007), as seen for “climbable” steps (Warren, 1984) or “crawlable” surfaces (Gibson et al., 1987).
2. Classical metrics are calculated, influenced by classical quantitative experimental psychology research: reaction times, “optimal” points, thresholds and relations between anthropometric data and actions possibility, etc. as to distinguish tendencies. Michaels (1988) suggested that “responses afforded in certain situations ought to be faster than responses not afforded” (pp. 231–232), and in Warren’s studies, if a step’s height is less than 88% of an individual’s leg length, then he can climb that step.
3. The experiments focus on the perception of affordances. The experimental environments are deliberately limited in terms of the number of objects available in order to direct the participant toward a particular affordance, and the associated action is simple, requiring only a single gesture. For example, in the studies by Gibson et al. (1987), the surface on which the young participants were invited to crawl was the only solution to move forward in the room, while Warren (1984) only proposed photos of a staircase, with no accompanying environment. This made it possible to focus on a specific (dispositional) affordance and see if it could be perceived, thus limiting bias.
4. Affordances are mostly considered in a dichotomous fashion: the action is either “possible” or “impossible,” as in the example of a stair being climbable or non-climbable by a particular individual (on this topic, Chouinard and Davis, 2016).

### 3.2 Contextual studies of affordances

We can now focus on the more contextual (or ecological) trend in affordance empirical research. Those studies are mostly based on considering culturally and socially situated affordances, which are not isolated but belong to landscapes, within ecological situations. As in the previous section we present the most representative ones, without aiming to be exhaustive.

#### 3.2.1 Studying affordances in context: an overview

Researchers are increasingly exploring childrens affordances through exploratory empirical studies. Bjørgen (2016) investigated physical activity in different kindergarten outdoor settings, using measures like actigraphs, questionnaires, and observations. She showed that physical and social affordances influence how children act, underlining that what the environment affords changes both possibilities to act (what children can do), and frequencies of behaviors (how often / regularly they act). Lerstrup and van den Bosch (2017) precisely observed and described self-initiated activities of young children during preschool hours, revealing that different places afford different behaviors. Sando and Sandseter (2020) collected data with systematic and random video observations of children free play in different outdoor environments, highlighting several types of affordances (other persons and animals, places, and objects), as well as inter-individual differences in what situations, objects and adults afford to different children. Later on, Moreira et al. (2023) studied how kindergarten affordances for physical activity depended both on what the situation afford and on preschoolers’ motor and social–emotional competences.

Other empirical studies have looked at psychopathology (Krueger, 2022). The important work in psychiatry by de Haan et al. (2013) provided a better understanding of the links between affordances and obsessive-compulsive disorder. They questioned 14 participants about their “everyday life” experience of the world, focusing on what had changed after a deep brain stimulation treatment on three main topics: “personal” (body, movements, perception, and skills); “social” (social interactions and relationships); “existential” (position relative to the disorder, perception and experience of time and freedom after the treatment). A main result is that there exists an evolving field of relevant affordances (i.e., solicitations) presenting three characteristics: a “width” (the number of available affordances, rarely reduced to one), a “depth” which takes up the temporal aspect of the evolution (the individual foresees, anticipates future changes), and a “height” dependent on that of each affordance (its intensity or force of attractiveness to the action). Such characteristics are themselves influenced by the concerns (motivations) of the patient, and by cultural and social dispositions.

In the field of sport, a number of studies have attempted to understand how athletes react to opportunities for action. Peker et al. (2023) used various behavioral measures to investigate how soccer players judged the maximum distance to kick the ball, and adjusted their type of kick between power and precision based on the situation and their experience. Esteves et al. (2011) showed that the position of the defender in basketball affords either to take a shot or drive toward the basket. In a review, Hettinga, Konings, & Pepping (2017) highlighted the importance of social affordances in competitive sports, showing how the presence or absence of an opponent changes athletes’ decision-making and pacing behavior. Other studies also aimed at understanding how individual-environment coupling takes place and enables performance based on action possibilities. Seifert et al. (2014) used a combination of objective (ratio of exploration / progression movement) and subjective (think-aloud verbalizations) data to study mountaineers exploring the surface of an icefall. They showed that the most efficient athletes exploit the constraints of the environment (here holes) to optimize movement. Rochat et al., 2019 used self-confrontation interviews to collect phenomenological data to investigate the impact of different hydration systems on trail runners’ possibilities of action, highlighting how typical runners improve performance by modifying their behaviors depending on the carrying system they use. Seifert et al. (2017) also studied the impact of the variability of interpersonal coordination and individual organization on rowing performance. They used self-confrontation interviews and sensors (acceleration, velocity) to show that behavioral and velocity perturbations are always experienced as meaningful by the rowers who adapt their movements to improve performance.

Other empirical work on affordances is also emerging in the field of art and architecture. An interesting example is the work on “The End of Sitting”^9^ by the Dutch architectural company RAAAF (Rietveld Architecture-Art Affordances) and the artist Barbara Visser. Withagen and Caljouw (2016) study sought to understand how such an atypical environment radically modified what the individuals were invited to do, and more particularly their behavioral habits (e.g., classically sitting at a desk). Two groups of 9 people had to work on an oral presentation in two environments (classic office vs. The End of Sitting). Several experimenters coded the video recordings of the activity focusing on the “localisation (of) the activity (categorized as reading the text, using the computer, talking, other, and not visible), and the posture” (p. 1022). Results show that individuals systematically chose working places based on their own body-structure and that what the environment affords disrupts behavioral habits.

#### 3.2.2 Characteristics of contextual experimental research

There are some common features that characterize these more contextual empirical works on affordances:

1. Affordances are understood in the broadest sense. They are general possibilities to interact with the world in several ways, be it mentally or physically.
2. Affordances are considered to be culturally and socially embedded. They are no longer “outside” of any cultural or social practice, and the studies highlight that these aspects have influence on them, such as codes associated with “places,” e.g., playfields being places to play (Sando and Sandseter, 2020)
3. Affordances belong to landscapes that should be studied. An affordance never appears “alone,” but is rather part of a complex landscape of possibilities for action and invitations to act. Studies in psychiatry show (e.g., de Haan et al., 2013; Dings, 2020) that people live in large landscapes of affordances and actually experience possibilities to act from moment to moment.
4. The methods used are exploratory in nature, aiming to better characterize affordances, by focusing on everyday and ecological situations, away from laboratories. These methods can provide an understanding of the influence of pathologies on the perception of the world, or of the relationship between the behaviors of athletes and their environment, a knowledge that may also apply beyond these fields.
5. The subjective experience of participants is considered: many studies go beyond mere measurements or observations, and add a phenomenological dimension to the empirical investigation. Recent work adopting a first-person perspective, such as that by Dings (2020, 2021), emphasizes individual self-referentiality, and not just bodily skills, in shaping perceptions of affordances.

### 3.3 Current empirical work is not enough to stabilize affordances

In the first part of this article, we emphasized the difficulty of finding a theoretical way out of the lack of consensus on affordances in order to stabilize a minimally shared concept. We then turned our attention to empirical work, and our review leads us to believe that if current empirical research clearly has benefits, it also has limitations, and may not be sufficient to overcome the theoretical issues at hand. Overall, there is a disarticulation between theoretical and empirical research that needs to be addressed in a principled way.

#### 3.3.1 Benefits and limitations of current empirical work on affordances

Both classical and contextual empirical works on affordances have shown it was possible to operationalize affordances and observe or measure them in various ways, highlighting some of their important dimensions.

Classical empirical studies have highlighted the importance of considering the sensorimotor aspects of affordances. Since Warren (1984), researchers (e.g., Petrucci et al., 2016) have defined operational ways of measuring affordances with quantitative indicators and proxies in the tradition of experimental psychology, and have brought to light invariants for humans, e.g., ratios defining when humans can “climb” a step, use a tool, etc. However, these approaches have often only considered affordances insofar as they trigger physical responses (Şahin et al., 2007). They do not consider them as invitations to act that can vary, keep away from theoretical extensions such as mental affordances, and do not consider phenomenological perspectives. Experimental situations are oriented toward sensori-motor responses (e.g., Barsingerhorn et al., 2012; Wokke et al., 2016), remain basic (climbing stairs, crossing gaps…), often oversimplified, and paradoxically non ecological. They allow very targeted results, but have also become somewhat standardized and largely repeated in the literature.

On their side, contextual empirical approaches have highlighted the interest of studying contextually situated affordances, and to focus on real and realistic contexts (e.g., Rochat et al., 2019; Krueger, 2022). In the vast majority of cases, they do not limit affordances to possibilities of action based solely on physiological qualities, and have incorporated recent theoretical extensions of affordances (e.g., de Haan et al., 2013 on enactive psychiatry; Withagen and Caljouw’s, 2016 for architecture). They have mostly used qualitative methods for measuring mental and social affordances in socio-cultural contexts, based on observation, ethnography or phenomenology. Despite rare studies using mixed methods to triangulate objective indicators and experiential data (Seifert et al., 2014, 2017, 2021), they have been heavily criticized for their difficulty to leverage quantitative data to measure affordances and to isolate variables to assess the weight of socio-cultural aspects, or for neglecting constituent aspects of affordances, such as sensory-motor ones (Segundo-Ortin and Heras-Escribano, 2023). Although they relate more to ecological issues than the traditional studies, empirical fields have nevertheless been limited to sport, psychopathology, or child development.

#### 3.3.2 On the disarticulation between theoretical and empirical work

As suggested by Lakatos (1970), scientific progress occurs through the development of research programs, which are characterized by empirical testing and the refinement of concepts based on the results of that testing, within communities that agree on their respective common grounds. In such a state of development, we could say that theoretical and empirical work are articulated, and work together. When it comes to current affordance research, it seems to us that, on the contrary, theoretical and empirical work are disarticulated. This second issue in affordance research prevents empirical work from playing its part and contributing to solve the first main issue, which is constitutive ambiguity of affordances.

This disarticulation comes from the fact that the concept was born without a clear associated methodological proposition. Indeed, Gibson’s proposition, already revolutionary in its proposal of direct perception, lacked an analytical method for identifying affordances (Oliver, 2005, p. 412). Unlike traditional empirical approaches of perception, taking seriously direct perception requires measuring the coupling between individuals and their environment, which presents conceptual and implementation challenges. As highlighted by Chong and Proctor (2020), there is no established method to measure an “ontological basis of affordances.” This means that, from the beginning, empirical researchers have been on their own to choose how they should operationalize affordances. It is then no surprise, as Heft pointed out in 2003, that there are so many different ways to study affordances: techniques, methods, and tools have proliferated, reflecting the diversity of contexts and approaches.

On the one hand, the strength of classical empirical research on affordances is that it proved the initial theoretical propositions, e.g., that possibilities of action are linked to physical abilities with invariants and that affordances are body-scaled. Yet, to our knowledge, those classical empirical explorations were hardly the source of theoretical evolution. Contextual empirical research, on the other hand, primarily aims at an exploratory understanding of the situations involved, focusing on accessibility, without seeking to validate theoretical propositions. These two types of empirical approaches do not have the same objectives, and seem hardly reconcilable today. Empirical research on affordances lacks a common ground, does not constitute a community, and cannot provide results that would allow us to settle on theoretical questions, for it does not fulfill its duty to provide evidence for theoretical concepts, nor does it aim at establishing stable definitions from empirical data.

This disarticulation between empirical and theoretical research on affordances may be self-perpetuating. The first reason lies in the difference between the pace of theoretical and empirical developments. As we have seen, theoretical frameworks have flourished in recent years, leading to a conceptual proliferation, while empirical research did not evolve much, often repeating the same studies or making minor adjustments to experimental protocols. Theoretical research seems to have rapidly outpaced the slower progression of empirical work, resulting in an inability for the latter to adequately contribute to conceptual development, exacerbating the disarticulation. The second reason is that empirical research on affordances seems devalued. Indeed, carrying out empirical work on affordances is complicated, especially when it comes to innovating in methodologies, and without the support of a community. Moreover, theorists, despite their productivity, seem to have lost interest in empirical data and do not seek validation of their proposals through empirical means. Overall, the differences in the pace of development and the lack of interest in empirical research feed off each other, widening the gap and discouraging further empirical work on affordances, thereby increasing the disarticulation.

To conclude, despite some real successes, the lack of common ground and the fragmentation of empirical work on affordances mean that current empirical research cannot help in settling on the lack of consensus on the concept. What is even worse, empirical and theoretical research appear disarticulated, and the only way out seems to try to rearticulate them.

## 4 Toward empirical methodologies for re-articulation

### 4.1 Re-articulating theoretical and empirical work on affordances

It is at the core of empirical work to enable theoretical intuitions to be anchored in real world data. On the one hand, the role of empirical data is to validate or invalidate theoretical propositions. When it comes to perception and action, theoretical propositions need to be verified empirically in real-life situations if they are not to remain mere hypotheses. This means that for example, for mental affordances to “exist,” empirical proofs are needed, or “measures” of some sort. Empirical work has the advantage of making it an obligation to settle on what is called an affordance, and how it will be measured, forcing theory to match minimal requirements for associated empirical work to be conducted. On the other hand, empirical work also aims at building invariants that can challenge theoretical work to progress further on or suggest definitions based on empirical data. Notably, empirical work on affordance helps to identify and explore similarities and differences in the ways individuals perceive them. If theoretical work can to some extent anticipate that there are invariants and differences, only empirical data can help settle and show precisely to what extent variability in sensory-motor abilities in humans, or in their expertise or mental health, change affordances perception.

For affordance research to reach the status of a research program and walk on both its theoretical and empirical feet, it seems necessary to re-articulate empirical and theoretical work.

If we consider the empirical to theoretical direction of re-articulation, empirical research would first benefit from being clear on the theoretical framework it uses and should precisely define the concept of affordance that is at stake in each study. Indeed, this will impact the type of collected data, the way to analyze it, and the conclusions that can be reached. For instance, if affordances are only related to sensory-motor skills, then empirical methodologies can focus on measuring behaviors or proxies of those sensory-motor abilities, whereas if affordances are cognitive or social, then including observation and interviews might be adapted. Secondly, empirical research should not deprive itself from taking into account recent innovative theoretical propositions, such as the socio-cultural aspects of affordances, or even the possibility of mental affordances. For us, this would mean using definitions and conceptualizations in line with those of the 4E cognition approach, with embodiment as an essential aspect reminding us of the sensory-motor character of affordances, but without being limited to it, and welcoming socio-cultural aspects of cognition.

Now, considering the theoretical to empirical direction of re-articulation, theoretical work should first take into account available empirical data, in order to avoid anchoring proposals in mere anecdotes or imagined experiments. Theoretical researchers should systematically try to leverage empirical data for theoretical refinements, integrating it in their elaborations, so as to justify, develop, or inform their proposals. They should also focus on systematically trying to operationalize these into empirical proxies, for instance by suggesting methodologies, indicators, or experimental designs adapted to the measurement of the kind of affordances they advocate. Such work on the theoretical side would anchor theoretical developments, and probably slow down theoretical inflation.

A crucial additional point is that most of the effort should probably be put on empirical work on affordances, which should be systematically promoted and sustained. Let us also note that all these proposals are related to community building, and should foster the emergence of one or more communities, consequent enough to conduct articulated theoretical and empirical work.

### 4.2 Prerequisites for empirical methodologies favoring the rearticulation of theoretical and empirical work on affordances

Here we propose a set of five prerequisites for building renewed methodologies for observing and measuring affordances that match theoretical advancements, and foster re-articulation.

#### 4.2.1 Prerequisite 1: provide an explicit and precise definition of affordances as possibilities to act

Providing an explicit and precise definition of what an affordance is seems to be a fundamental starting point for any author designing an affordance-related empirical methodology. This definition should draw inspiration from the Gibsonian notion of possibility to act, and be related to a direct perception theory. This effort seems crucial to move forward, because providing explicit definitions will foster shared understanding of affordances, as well as the accumulation of comparable empirical data. A recent definition of affordances by Kimmel and Groth (2023) as “recognizable pointers to action opportunities in the ecology” may serve as a starting point, but other definitions might be used as well.

#### 4.2.2 Prerequisite 2: compare individual perceptions of affordances

Studying inter-individual variations in affordance perception is important in order to identify factors likely to influence it, or even invariants. Classical experiments have already done so, as in Warren’s famous work on staircases, or for the passability under a barrier studied by van Der Meer (1997), but this focus on inter-individual differences must be systematically encouraged. Recent examples for more qualitative studies can be found in the works of Seifert et al. (2014) on ice climbing or de Haan et al. (2015) for psychopathology. Some example characteristics useful for comparing participants might be their levels of expertise (eg. novice vs. expert), their emotional or mental states (eg. depression vs. OCD), their physiological properties (eg. height, thirst), their concerns,^10^ etc.

#### 4.2.3 Prerequisite 3: study lived experience of affordances

As highlighted by the theoretical proposals on sollicitations, notably van Dijk and Rietveld’s (2017) *Skilled intentionality Framework*, studying the phenomenology of subject-environment interactions from an empirical perspective seems necessary. As Heft (2003, p.151) has it, “perceiving the affordances of our environment is, if you will, a first-order experience that is manifested in the flow of our ongoing perceiving and acting.” Empirical research on affordances should then also focus on the way specific individuals are specifically “invited” to respond to the various possibilities to act they are faced with, and give priority to certain, while completely ignoring others. The empirical study of the lived experience of affordances is currently underrated (Dings, 2018, 2020), with only a few pieces of work trying to clarify individual perspectives of participants (eg. de Haan et al., 2013). Such type of study is also necessary for mental or higher cognition affordances which cannot be grasped without asking people themselves.

#### 4.2.4 Prerequisite 4: study affordances in ecologically valid contexts

Recent research has shown that the fact that affordances are multiple, and belong to rich landscapes must be taken into consideration at the empirical level (Rietveld and Kiverstein, 2014; Bruineberg et al., 2021). It seems no longer possible to neglect the fact that affordances are inscribed in a context, and to consider ecological validity as a central point of empirical research. This means that researchers should (1) study affordances in everyday life situations, even in laboratory design or settings, and (2) design situations complex enough for participants to be faced with and experience landscapes of affordances.

#### 4.2.5 Prerequisite 5: track affordances by collecting multiple types of proxies and studying them using mixed-methods

Catching on the spot the various possibilities of action one is faced with and experiences, whether these actions are realized or not, necessitates multiplying the proxies that may be of help. Be it in the laboratory or in real-life situations, empirical studies of affordances should be based on collecting various types of data, enriching classical physiological and behavior tracking with lived experience data collection, and on analyzing these data using various indicators and metrics, making room for inter-individual differences. Some of those proxies should be experience-focused, even in experimental settings. We propose that behavioral data represents an additional avenue of research into the “objectifiable” elements of affordances, in particular by integrating physiological aspects (such as eye-tracking).

### 4.3 A framework for designing empirical methodologies

Let us discuss the practical consequences of using our 5 prerequisites, and propose a framework for designing associated empirical methodologies.

Prerequisite 1 forces one to settle on a working definition of affordance inline with the possibility-to-act perspective, and to discuss how it relates to Gibson’s work and to direct perception. Only a few pieces of empirical work have explicitly done so, good examples can be found in Petrucci et al. (2016) for a sensorimotor approach, or in Rietveld et al. (2013) for social affordances. To discuss precisely the empirical consequences^11^ of chosen definitions is also necessary. For instance metrics can be related to counting effective actions, asking for go/nogo evaluations, measuring “action readiness” with electromyography, searching for invitations to act in the experience, etc.

Prerequisite 2 highlights the importance of comparing individuals to understand affordance perception. To do so, researchers must choose the characteristics they are interested in. These can be induced (e.g., stress or hunger) or not (e.g., physical, socio-demographic, pathological, or personal concerns). They should also define metrics about affordance perception that will be used to compare the groups with the different characteristics, and choose the methods to assess similarities and differences.

Prerequisite 3 underlines the need to elicit individual experiences of affordances. Various methods to access phenomenological data can be used, ranging from questionnaires during or after a task (“Are you aware of the hammer?,” “During the task, did you consider using the hammer?”), to dedicated interviews such as micro-phenomenological (Petitmengin, 2006) or self-confrontation (Nielsen, 2006) interviews.^12^ Analyzing lived experiences can follow top-down approaches if one is using questionnaires or already has established categories; or more grounded ones, with more or less structured variants of thematic analyze, e.g., analyzing globally the categories of the experiences of a group of participants, or modeling diachronically and synchronically each interview transcription (Valenzuela-Moguillansky and Vásquez-Rosati, 2019).

Prerequisite 4 states that experimental designs should foster the development of studies that take place in ecologically valid contexts. Yet, empirical research also calls for sufficiently controllable situations, and we can at least distinguish three possibilities for doing so. 1/ Modifying an existing situation in natural settings, for instance, adjust certain variables in a real situation (e.g., change the sizes, colors, arrangements of objects in space) to observe any impact; 2/ Creating experimental situations and controlling their parameters, for instance building artificial virtual reality environments that mimic everyday life situations and being able to parameters them (e.g., change the position of the oven in a kitchen, swap surrounding people in public transportation); 3/ Observing and studying everyday situations as to understand how affordances might change in specific situations that have not been influenced by researchers.

Prerequisite 5 focuses on the fact that empirical methodologies have to include multiple proxies and use mixed-methods to study affordances. As we have seen, quantitative indicators related to behavioral (reaction time, possibility of performing the action, eye-tracking), or physiological (electroencephalogram, electromyogram, blink rate, HRV) dimensions have long been used. Qualitative indicators collected from interviews or questionnaires that capture how participants experience affordances and their solicitations as possibilities of action have been featured in more recent approaches. It is noteworthy that those approaches belong to what is known as “experimental phenomenology,” developed by pioneers such as Michotte^13^ and Katz,^14^ and also present in Gibson’s work, as pointed out by Heft (2013). However, the claim of prerequisite 5 is that empirical research, as phenomenologically grounded as it may be, should continue using metrics based on behaviors and not reject sensory-motor aspects of affordances. Articulating various perspectives with mixed-methods seems a good way to do so. Much work is now needed on how to articulate such data in a principled way. Data triangulation can for example be carried out by overlaying behavioral metrics with first person perspectives on affordances to understand how people manage to deal with multiple affordances and selectively respond to one [see *the affordance competition hypothesis* - Cisek and Kalaska (2010)], or how individual concerns influence affordance perception [see *Meaningful affordances* - Dings (2021)].

Those practical consequences of our prerequisites can be merged in the framework shown in Table 2, and give us a design space for creating empirical methodologies by choosing amongst various possibilities.

**Table 2:** A framework for building empirical studies of affordances.

Prerequisite	Possibilities
**(1) Precisely define affordances as possibilities to act**	Defining affordances in relation to Gibson’s work and direct perception - Physical affordances, Social affordances, Mental affordances… Discussing the implications of the chosen definition with regards to operationalization and possible empirical metrics, based on - Body, psychological state, experience, etc. - Environmental variables
**(2) Focus on individual perception of affordances**	Defining the characteristics that are addressed by the study - Expertise level, psychopathology, personality, cognitive ability… - Induced / non-induced Defining comparison methods - Categories, statistics, tests, etc.
**(3) Study lived experience**	Collecting lived experience data can make use of - Offline and/or inline questionnaires - Micro-phenomenological interviews - Self-confrontation interviews - … Analyzing lived experience - Top-down or bottom-up - Individual or group level - Thematic or structured modeling - …
**(4) Use ecologically valid contexts**	Ecological context can come from - The modification of an existing situation - The creation of an experimental situation in VR - The study of an everyday situation
**(5) Collect multiple types of data and use mixed-methods**	Articulation of data can concern any mix of - Behavior and/or physiological data - With experience data

In the remainder of this article we will show that two studies we have already seen follow our requirements, and rapidly describe some other studies that can be designed from our framework.

In our first example study, de Haan et al. (2013) used an explicit enactive-based understanding of affordances (1) as possibilities of action, focusing on the dynamics between the individual and the world. They used semi-structured interviews with personal-, social- and existential-related open questions to phenomenologically analyze individual experiences and their differences (2–3) studying on everyday situations (4) of OCD patients with deep brain stimulation treatments. Yet they did not try to use other affordance-related data (5) within mixed-methods. Our second example is the study by Seifert et al. (2014), who primarily worked on clearly defined sensori-motor affordances (1) in ice-climbing, focusing on individual differences (2) between experts and non-experts. They collected lived experience data (3) using self-confrontation interviews of experts and beginners having climbed a 30 m icefall in a natural setting, which they analyzed thematically (4). They assessed climbing affordances using both behavioral metrics (ratio between exploratory movements and performed actions), and lived experience data (5).

The two studies we just mentioned are good examples of works that can participate in re-articulating empirical and theoretical work on affordances. They roughly follow the requirements and the possibilities we underlined in our framework. Yet this framework can also act as a design space and help designing empirical studies by picking various possibilities within the requirements. Without exhaustivity, here are a few interesting methodologies that emerge:

- Studying mental and sensori-motor affordances by using self-confrontation interviews and electromyography (EMG) in a controlled context such as a sport training camp. More specifically, the study could focus on identifying and describing decision-making moments in a ball game, when several possibilities are available for passing the ball. This could be done by collecting precise verbal descriptions of the experiences of participants watching ball-passing moments on first-person videos (what they saw, any reasoning they had, etc.), categorize them thematically, and triangulate the themes and moments with action readiness as measured from EMG data. Such kinds of study already exist in sports, but they do not use affordances as a theoretical framework.
- Studying affordances related to social interactions in virtual reality, using online questionnaires and eye-trackers to understand how people perceive social affordances in various social contexts (office, party, etc.). A VR environment could be created in which participants have to carry out tasks that need social interaction. The study could focus on understanding how individuals consider with whom to talk to at specific moments, using both visual cues from eye-tracking data, and answers from questionnaires people would fill in soon after the interaction has finished.
- Studying how different students perceive mental affordances related to what the teacher says and the activities they carry in class, using behavioral records and micro-phenomenological interviews. A focus could be put on the way the teacher instructions throughout the activity affect the way participants conduct their reasoning and make decisions in a mathematical exercise. This exploratory study could imply live or post- observation of the activity, so as to identify moments around which to interview students, and carry-out precise modeling of lived experience of how thinking unfolds.
- Studying mental and sensori-motor affordances for psychiatric patients in artificial settings mixing first-person interviews and behavioral data tracking to understand how mental health (e.g., depression) influences the perception of possibilities of actions in those environments. One could for instance measure how the same object (a glass, a phone, etc.) affords different actions depending on the mental state of the patient. This may be related to the work of Bague and Laurent (2023) on the differences in perceived reachability between depressive and non depressive patients.
- Studying the dynamics of solicitations / invitations to act using controlled VR environments where participants carry their tasks, collecting and articulating behavioral and lived experience data. This is the case in one of our projects where we study how normal and anxious patients differ in behavior when searching for an item in an everyday situation (make a cake in a VR kitchen), articulating such external data with a modeling of the experiences they had of different solicitations in the course of the activity.

These examples are by no means final, and we hope the framework can encourage empirical researchers to participate in re-articulating theoretical and empirical work on affordances, multiplying the fields of study and the methods.

## 5 Conclusion

Theoretical developments on affordances have multiplied, and the notion has become a gold mine for academic scholars, who “get to write learned articles about the true meaning of the term” (Norman, 2008, p.19). As a result, the concept lacks stability, and may be at risk of losing scientificity. However, more than 40 years since Gibson’s seminal work, affordances remain central to post-cognitive theories and are extensively applied across various research domains. Their potential for application is vast, and we may be witnessing “the coming of age of ecological psychology” (Bruineberg et al., 2023). Empirical work has been split between classical empirical laboratory work and contextual-ecological studies. It remains scarce, hardly catches up with theory, and cannot play its role in scientific development, as it lacks articulation with theoretical work. This is why the re-articulation of theoretical and empirical work on affordances seems necessary to chart a more productive course that will enable the concept to make healthy progress towards its promises. To achieve such a goal, focusing on empirical work is what affords us the most. This can be done by multiplying empirical studies on affordances, and by enhancing the associated empirical methodologies. We believe our framework can contribute to this.

## Author contributions

RM: Writing – original draft, Writing – review & editing. YP: Writing – original draft, Writing – review & editing.

## Footnotes

^1^For a critical response see Fodor and Pylyshyn (1981).

^2^We deliberately exclude the affordance “as a resource” approach proposed by Reed (1997), which has not gained traction since 2000.

^3^In proposing this notion of “potentiality” Rietveld and Kiverstein (2014) draw on Chemero (2011), pp. 149–150) and Heft (2003, p. 132).

^4^The term, borrowed from Gestalt psychology, appeared for the first time in connection with affordances in a publication by Dreyfus and Kelly (2007, pp. 52).

^5^Costall (1995, p. 477) notes that affordances put “meaning back into the world”.

^6^Affordances have also been called in and extended even further. For example, in morality research, Hampson et al. (2021) suggested that individual choices correspond to the detection and selection of “moral” affordances that allow one to remain in an optimal decision-making zone. Harré (2014) also used the term affordance to replace the Popperian concept of propensity in chemistry or quantum physics (Brock and Harré, 2016). These extensions may be theoretically coherent on their own, they nevertheless radically differ from the initial Gibsonian understanding, and even from most of the remobilizations we have evoked.

^7^The term “classical” does not imply any judgment on the value of the studies, we use it to refer to the first wave of studies on the perception of affordances, which represent the seminal studies on this topic.

^8^For the sake of clarity, we prefer to use the adjective “contextual” rather than “ecological,” which may nevertheless be more appropriate. Indeed, it is necessary to distinguish between an “ecological” approach to empirical research which studies real-life situations and ecological psychology as a disciplinary field.

^9^Another setting in this line is the “Still Life” environment composed of 4 vertical plates in a room, moving simultaneously back and forth on linear paths. As one walks in the room, one comes across the trajectory of plates in an always changing environment that unpredictively changes one’s possibilities to act (see Bruineberg et al., 2021).

^10^Dings (2021) suggests that some affordances become meaningful because we have specific concerns related to motivation.

^11^Let us also remark that if one defines an affordance as a possibility of action, then one should also assume the consequence that even only considered or simply refrained actions are to be taken into account.

^12^For a review of first-person approaches to studying experience, see Lumma and Weger (2023).

^13^Albert Michotte (1881–1965) was a key figure in the field of experimental phenomenology. Michotte’s approach focused on studying the conscious experiences and subjective perceptions of human subjects, particularly in relation to visual perception and the interpretation of visual stimuli.

^14^ David Katz (1884–1953) was a pioneering experimental psychologist who conducted extensive research in the early years of psychology as an independent scientific field. His work primarily focused on color vision and touch, and he employed a unique approach that he referred to as the “phenomenological method.”
